# The first Malay database toward the ethnic-specific target molecular variation

**DOI:** 10.1186/s13104-015-1123-y

**Published:** 2015-04-30

**Authors:** Hashim Halim-Fikri, Ali Etemad, Ahmad Zubaidi Abdul Latif, Amir Feisal Merican, Atif Amin Baig, Azlina Ahmad Annuar, Endom Ismail, Iman Salahshourifar, Ahmad Tajudin Liza-Sharmini, Marini Ramli, Mohamed Irwan Shah, Muhammad Farid Johan, Nik Norliza Nik Hassan, Noraishah Mydin Abdul-Aziz, Noor Haslina Mohd Noor, Ab Rajab Nur-Shafawati, Rosline Hassan, Rosnah Bahar, Rosnah Binti Zain, Shafini Mohamed Yusoff, Surini Yusoff, Soon Guan Tan, Meow-Keong Thong, Hatin Wan-Isa, Wan Zaidah Abdullah, Zahurin Mohamed, Zarina Abdul Latiff, Bin Alwi Zilfalil, In alphabetical order, members of the Malaysian Node of the Human Variome Project

**Affiliations:** Department of Pediatric, School of Medical Sciences, Universiti Sains Malaysia, Kubang Kerian, 16150 Kelantan, Malaysia; Human Genome Center, School of Medical Sciences, Universiti Sains Malaysia, Kubang Kerian, 16150 Kelantan, Malaysia; Department of Hematology, School of Medical Sciences, Universiti Sains Malaysia, Kubang Kerian, 16150 Kelantan, Malaysia; Department of Ophthalmology, School of Medical Sciences, Universiti Sains Malaysia, Kubang Kerian, 16150 Kelantan, Malaysia; Faculty of Medicine, Universiti Sultan Zainal Abidin (UniSZA), 20400 Kuala Terengganu, Terengganu Malaysia; Molecular Medicine Cluster, Biomedical Center, Faculty of Medicine, Universiti Sultan Zainal Abidin (UniSZA), 20400 Kuala Terengganu, Terengganu Malaysia; Centre of Research for Computational Sciences and Informatics in Biology, Bioindustry, Environment, Agriculture & Healthcare, University of Malaya, 50603 Kuala Lumpur, Malaysia; Institute of Biological Sciences, University of Malaya, 50603 Kuala Lumpur, Malaysia; School of Biosciences and Biotechnology, National University of Malaysia, 43600 Bangi, Selangor Malaysia; Department of Biomedical Science, Faculty of Medicine, University of Malaya, Kuala Lumpur, Malaysia; Department of Parasitology, Faculty of Medicine, University of Malaya, Kuala Lumpur, Malaysia; School of Health Sciences, Universiti Sains Malaysia, Kota Bharu, Kelantan Malaysia; Oral Cancer Research Coordinating Centre, Faculty of Dentistry, University of Malaya, Kuala Lumpur, Malaysia; Faculty of Biotechnology & Biomolecular Sciences, Universiti Putra Malaysia, Serdang, Selangor Malaysia; Department of Pediatrics, Faculty of Medicine, University of Malaya, Kuala Lumpur, Malaysia; Department of Pharmacology, Faculty of Medicine, University of Malaya, Kuala Lumpur, Malaysia; Department of Pediatrics, Faculty of Medicine, Universiti Kebangsaan Malaysia Medical Centre (UKMMC), Kuala Lumpur, Malaysia

**Keywords:** Malaysian Node of the Human Variome Project, Ethnic-specific molecular variation database, SNPs, CNVs, Disease genes and their products

## Abstract

**Background:**

The Malaysian Node of the Human Variome Project (MyHVP) is one of the eighteen official Human Variome Project (HVP) country-specific nodes. Since its inception in 9^th^ October 2010, MyHVP has attracted the significant number of Malaysian clinicians and researchers to participate and contribute their data to this project. MyHVP also act as the center of coordination for genotypic and phenotypic variation studies of the Malaysian population. A specialized database was developed to store and manage the data based on genetic variations which also associated with health and disease of Malaysian ethnic groups. This ethnic-specific database is called the Malaysian Node of the Human Variome Project database (MyHVPDb).

**Findings:**

Currently, MyHVPDb provides only information about the genetic variations and mutations found in the Malays. In the near future, it will expand for the other Malaysian ethnics as well. The data sets are specified based on diseases or genetic mutation types which have three main subcategories: Single Nucleotide Polymorphism (SNP), Copy Number Variation (CNV) followed by the mutations which code for the common diseases among Malaysians. MyHVPDb has been open to the local researchers, academicians and students through the registration at the portal of MyHVP (http://hvpmalaysia.kk.usm.my/mhgvc/index.php?id=register).

**Conclusions:**

This database would be useful for clinicians and researchers who are interested in doing a study on genomics population and genetic diseases in order to obtain up-to-date and accurate information regarding the population-specific variations and also useful for those in countries with similar ethnic background.

## Findings

Studying the variations in the human genome represents new horizons in genetic research, which would help to reduce health problems and develop new strategies towards designing better diagnostic and preventive approaches. Many general and locus specific mutation disease databases have been established [1-4]. It is also well known that different ethnic backgrounds may have different disease-causing mutation(s) and variation(s). Therefore, population specific databases are beneficial not only for future surveys, but also for those conducting studies in the etiology of genetic disorders and distribution of the mutations.

With the advent of the SNPs-based microarray, a huge amount of data was added to the growing body of knowledge for each specific disease. The role of SNPs in the etiology of the genetic disorders, particularly in complex traits has been well accepted [5-7]. The SNPs may contribute to the genetic disorder or they may be in linkage disequilibrium with other casual variants and mutations. Different genetic backgrounds may have different susceptibility to the haploid in the sufficiency of variants or mutations. In addition, the variable prevalence of the same genetic disorder among various populations suggests the contribution of different individual genetic variants for each ethnic background. Therefore, establishment of a country-based molecular variation database will facilitate the accessibility of researchers to the genetic differences between various ethnic groups and ancestries. Such database will help to trace the population diversity, history and disease susceptibility for each sub-population [8]. MyHVP is a continuation of the Human Variome Project (HVP) which was detained in Beijing Meeting report at 2011 also, could be considered as an initial report in the updating of the Malaysian Node as an ongoing process. Therefore, MyHVPDb does not create the new databases; however, it will be continuously updating the database to be more comprehensive and reliable. In consequence, the MyHVPDb attempts to address the lack of coordinated effort in collecting and compiling genomic variations and common Mendelian disorders that might be associated with the multi-ethnic Malaysian population.

### Ethnic background and common disorders in Malaysia

**Figure 1:**
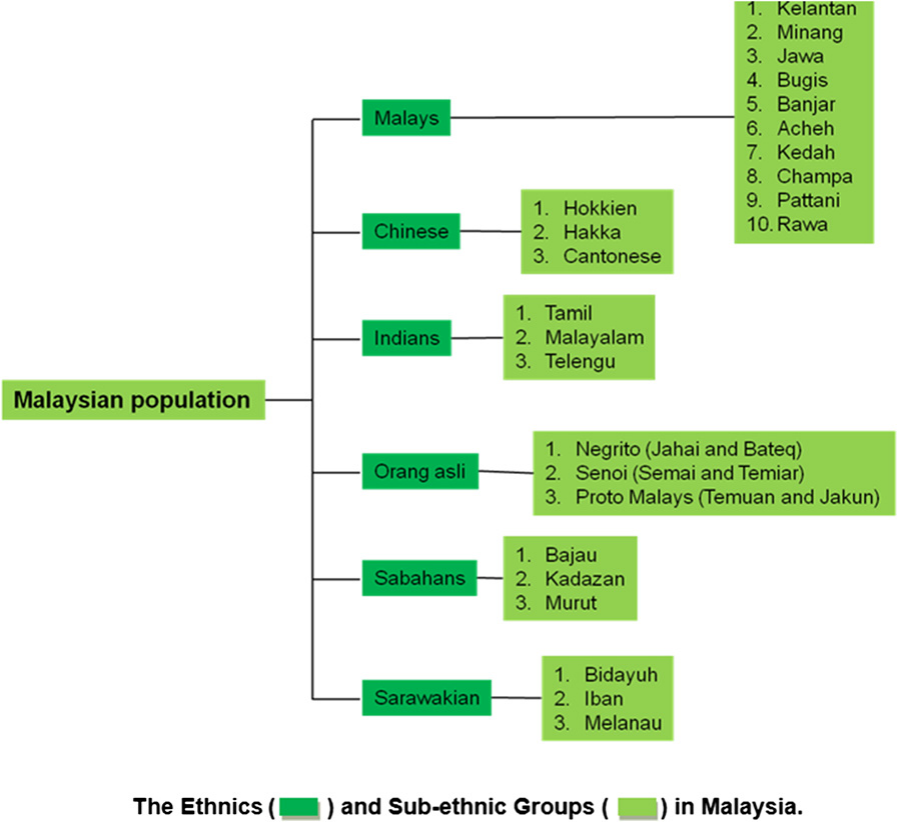
The Ethnics(rectangle green) and Sub-ethnic Groups (rectangle yellow green in Malaysia.

Malaysia is located in Southeast Asia and separated into two regions by the South China Sea, namely Peninsular Malaysia and East Malaysia (the latter is composed of Sabah and Sarawak). It has land borders with Thailand, Indonesia and Brunei also, maritime borders with Singapore, Vietnam and the Philippines. Malaysia is a multi-ethnic country with three major ethnic groups (Malay, Chinese and Indian); aborigines (Orang Asli which consist of Proto Malays, Negrito and Senoi); Sabahans and Sarawakian (Major sub-ethnic groups of Sabahan are Kadazan/Dusun, Bajau and Murut; followed by urban, Bidayuh and Melanau respectively as Major sub-ethnic groups of Sarawakian). Each ethnic group is further divided into sub-ethnic groups, representing the existing diversity of Malaysian population (Figure 1). The total population is 28.3 million, where Malays comprised 63% of the total population, followed by Chinese (28%), Indians (8%), and other ethnic groups (1%) [9].

According to the Ministry of Health Malaysia, heart related disorders were the most common causes of death (16.09%), followed by Septicaemia (13.82%) and Malignant Neoplasms (10.85%) [10]. Regardless of ethnic groups, breast, colorectal, lung, cervix and nasopharynx cancers were the five most common malignancies among the population of Peninsular Malaysia. Leukemia is the most common cancer among children below 14 years old. Mendelian genetic disorders such as thalassemia [11], Duchenne muscular atrophy [12], spinal muscular atrophy [13], retinoblastoma [14], G6PD deficiency [15] and orofacial clefts [16] are also relatively common in the country.

The different ethnic group is known to have risks of certain diseases, for example thalassemia in Southeast Asia, sickle cell anemia in Negroid population, and hemochromatosis in Jews [2]. Genetically, spectrum of mutations differ according to different ethnic for the same respected gene and disorder. Therefore, the MyHVPDb intentionally includes mutational data for communal gene disease among Malaysians ethnics.

## Materials and methods

### Database construction and implementation

**Figure 2:**
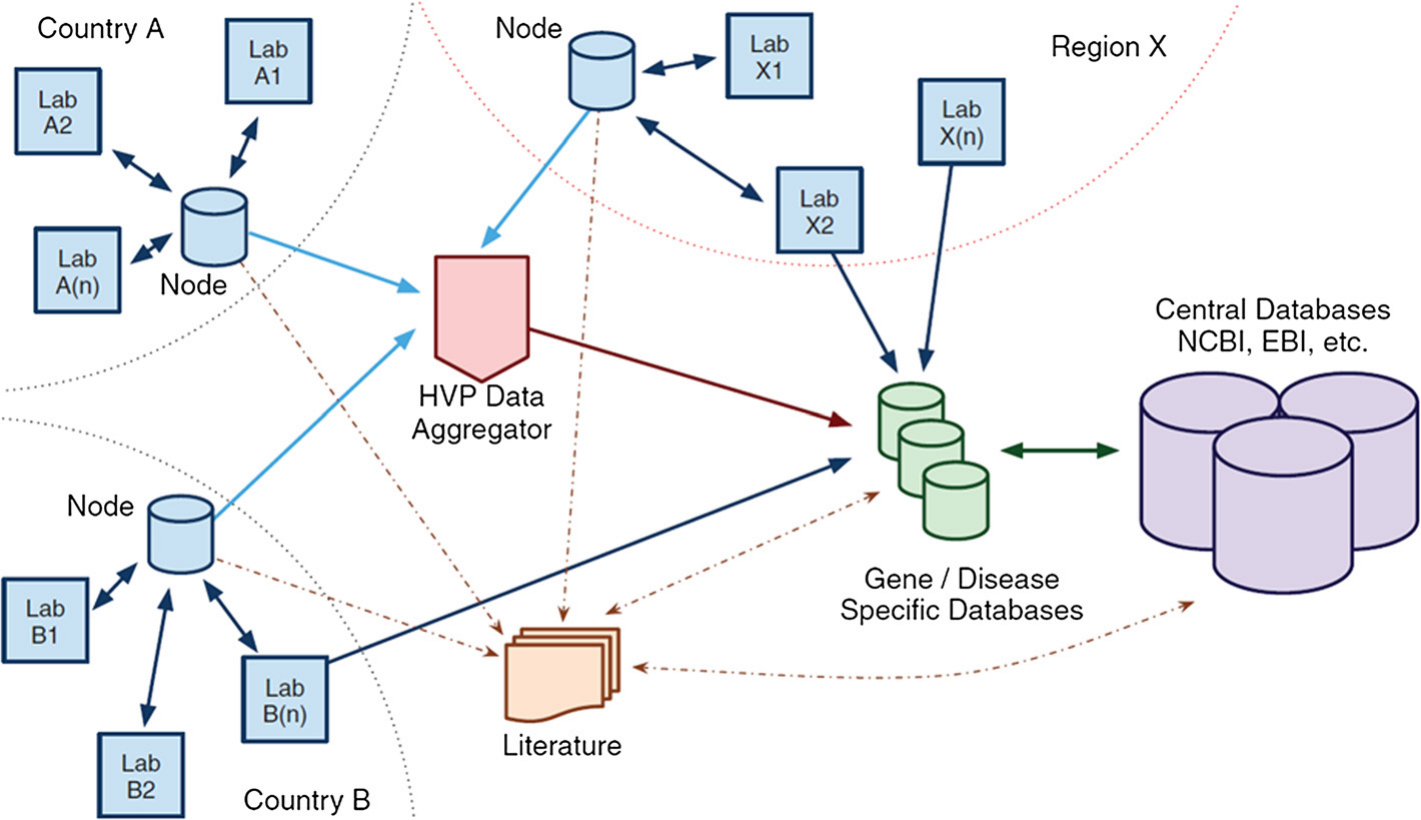
The Proposed Data Collection Architecture of the Human Variome Project “Reprinted by Permission from Al Aama et al. [17].

In order to ensure that the stored data sets in Malaysian Node of the Human Variome Project Database (MyHVPDb) can be effectively shared, core elements of the data, followed by the standard nomenclature similarly adopted by international databases and the standard HVP database architecture were recruited (Figure 2). Data sharing was done automatically using standardized descriptors and controlled vocabularies via the HVP Data Aggregator. Data Aggregator will allow HVP Country Nodes to automatically share existing data for possible with every gene/disease specific database in the Human Variome Project. All of this sharing was accomplished through a single data link to the aggregator, thus HVP Country Nodes do not need to establish and maintain the links to every database.

**Figure 3:**
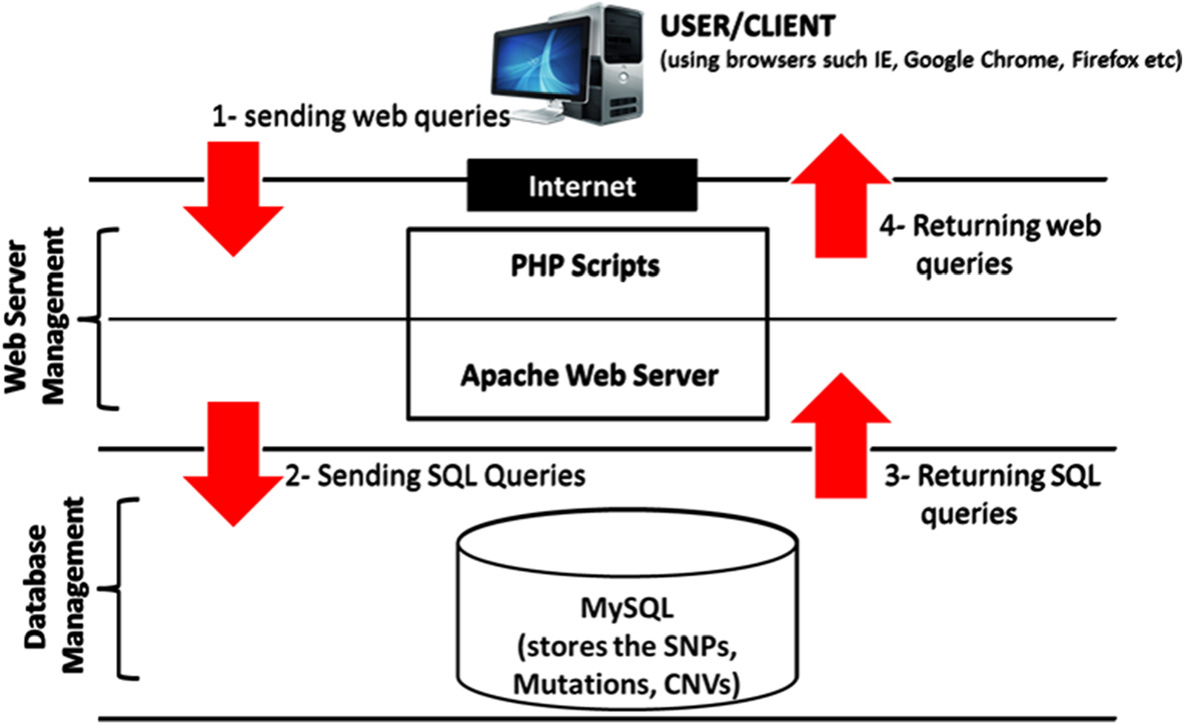
Schematic Representation of the Architecture of Malayasian Node of the Human Variome Project Database (MyHVPDb).

Additionally, the content and structure of MyHVPDb follows guidelines given by NCBI SNPs Database (http://www.ncbi.nlm.nih.gov/projects/SNP/). The overall design of MyHVPDb was based on three-tier architecture model (client, web and database) as shown in Figure 3. The database was developed using Hypertext Markup Language (HTML), Cascading Style Sheet (CSS), Java Script, Hypertext Preprocessor (PHP) and Structured Query Language (SQL). PHP scripts were used as a Common Gateway Interface (CGI) for sending and receiving data between the front end user/client and the database server. To query data, users need to enter or choose a keyword such as SNP ID or chromosome number. The Apache web server will then transform the query into SQL for it to be requested from MySQL database.

### Database access

The MyHVPDb can be accessed through http://hvpmalaysia.kk.usm.my which consists of the database administrator and user portal. The user portal has been divided into two status of privilege access: advanced and basic, whereby basic users will have some limitations in the scope of available data for viewing (Table 1).

**Table 1:** **Accessibility for MyHVPDb database according to the categories, followed by the number of SNPs among six Malay sub-ethnicities**

**Feature**	**User**		**Administrator**
	**Basic**	**Advance**	**-**
Access to SNP/Mutation/CNV	Limited access	Full access	Yes
Registration and login required	Yes	Yes	Yes
Insert and edit data	No	No	Yes
Data export	No	No	Yes
**Malay sub-ethnicity**		**Total SNPs**	
Champa		57,702	
Kedah		57,536	
Kelantan		53,143	
Banjar		40,585	
Jawa		42,345	
Bugis		40, 406	
Total		291, 718	

A new user must register before being allowed to access the databases. Should there be any doubt regarding the information given in the registration fields, the database administrator will contact the user through email to request further information. Upon approval by the database administrator, the new user will be allowed to access the database as preferably requested.

### Querying the database

For the SNP database, there are two methods of searching the SNPs datasets, either by ‘SNP ID’ or ‘Chromosome Number’. It is easier to search the database using ‘Chromosome Number’ for the advanced level user because it provides information on the sub-ethnicity group in addition to the chromosome numbers. By using the specific search, it is easier to retrieve accurate results of the SNP associated with a particular sub-ethnic group. However, for the basic level user, no information about the sub - ethnicity group is made available.

All outputs from the search result either by ‘SNP ID’ or ‘Chromosome Number’ will be linked to the NCBI SNP database based on SNP ID in order to assist users in obtaining further information pertaining to the DNA sequences. The purpose of connecting to the NCBI SNP database is to ensure a high quality of the stored data and its reliability.

### Data submission

Submissions of new data could be carried out using the submission form provided on the website. The completed form should be sent to: 1mhgvc.hvp.secretariat@gmail.com.

We applied for the ethical approval through the respected organizations based on the Malaysian Human Variome Project: USMKK/PPP/JEPeM [231.3.(060] and Thalassemia project, with two ethical approval, (Ministry of Health: NMRR-12-980-13829 and USM: FWA No. 00007718; IRB No. 00004494).

## Results and discussion

### Database content

**Figure 4:**
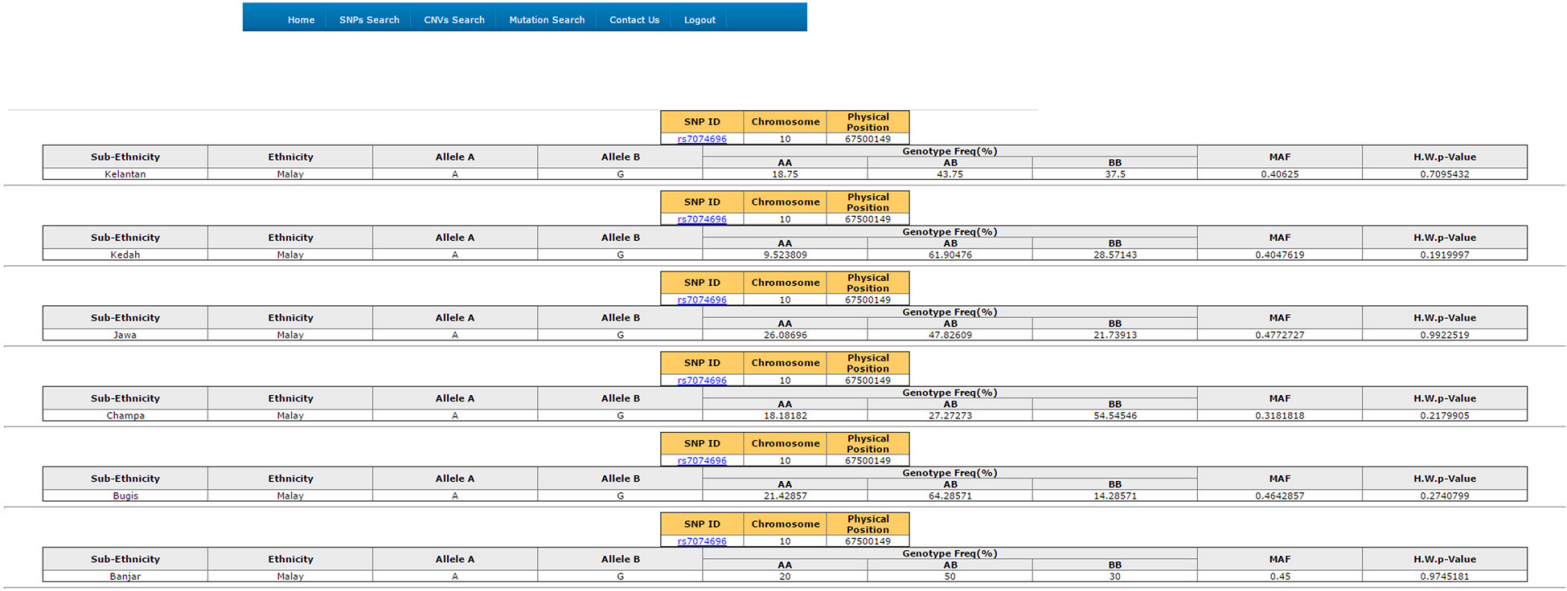
The format of SNP ID in MyHVP SNP Database and its supplementary information within each sub-ethnicity determined by the Minor Allele Frequency.
ii. **CNV database**
  This component of MyHVPDb provides the copy number variation (CNV) and disease gene(s). This database currently consists of CNV of the *SMN*2 gene associated with spinal muscular atrophy in Malaysia. We are analysing the CNVs based on healthy individuals who belong to different ethnicities and sub-ethnicities.
iii. **Mutational database**
  This database stores the data regarding to the specific gene(s) and mutation(s) that are related to Malaysian common diseases. It now consists of 143 mutations from 16 genes and 13 diseases. Some of the disease group are cancer, enzyme deficiency, infectious diseases, metabolic disease and hematological diseases. These diseases have been extensively studied with regards to its susceptibility and association with Malaysian population’s genetic makeup in order to provide a robust platform for diagnosis to enhance research opportunities and treatment development.

MyHVPDb is categorized into three main components:

i. **SNP database**
  The SNP databases content SNP data derived from Malaysian ethnic. During initial development of MyHVPDb, SNPs datasets of six Malay sub-ethnic groups; (Kelantan Malays, Minang Malays, Jawa Malays, Banjar Malays, Kedah Malays and Bugis) were stored (Figure 1).
  These SNPs data were obtained from the genotyping of 101 healthy Malay individuals, i.e. Champa (N = 12), Kelantan (N = 18), Banjar (N = 12), Bugis (N = 14), Kedah (N = 25) and Jawa (N = 20) sub-ethnic. The criteria of these individuals were based on: (1) Ancestry: having at least three generations of the same sub-ethnic including (2) Parentage: both parents were from the same Sub-ethnic (3) Religion: Muslim (4) Language: communicate daily using local Malay dialect and (5) Health status: healthy individual.
  Genotyping was performed using the 50 k Affymetrix chips, SNPs from this platform were compatible with the NCBI reported SNPs.
  The current number of SNPs for the Malay sub-ethnic group is 291,718. The highest numbers of SNPs among these six Malay sub-ethnicities were found in Malay Champa (19.78%) while the lowest numbers of SNPs were found in Bugis (13.85%) data sets (Table 1).
  From all these SNPs, those with known SNP ID registered in the NCBI SNP database is compared for genotype specificity. Basic information such as chromosome number, physical position, ethnicity, Allele A, Allele B, genotype frequency, Minor Allele Frequency (MAF) and H.W.P Value are provided. Currently, the data from other ethnic groups are undergoing the analysis and will be soon available in the database.
  Search based on SNP ID will display information including sub-ethnicity by combining in a single page (Figure 4). Therefore, it would be easier for users to compare the SNP information of each sub-ethnicity. Comparing the allele frequency of the genome wide SNP and genetic relationships among the population can be reconstructed by providing important clues about how humans adapted to changing climatic and nutritional environment. Moreover, some of these adaptations have important medical relevance, as they can be linked to differential disease susceptibility.

These Malaysian mutational data were extracted from published scientific articles of studies conducted on the Malaysian population. The available information in the database is Gene Name, Disease, Position of Mutation, Nomenclature, Type of Mutation, Locus of Mutation, Description of Mutation and Effect of Mutation.

## Conclusion

To date, the members of the MyHVP consist of 68 individuals from 12 Malaysian Universities and academic institutions. MyHVP also received support from professional Societies including the Genetics Society of Malaysia, the Malaysian Society of Human Genetics, the Medical Genetics Society of Malaysia, and the Malaysian Society of Bioinformatics and Computational Biology. Since its launch 3 years ago, MyHVP has attracted an increasing number of researchers and scientists to participate in this project across Malaysia. We have succeeded in developing an online database for the mutation of genes related to diseases discovered in Malaysia and also the Malay whole genome SNP database. MyHVP marks a new horizon of genetics activities in Malaysia and provides the specific database that relates to the Malaysian population. This database will be a useful resource for others countries with a similar ethnic groups.

The aim of this project would be the development of Southeast Asia (SEA) node of HVP, (HVP SEA node) which is expected to communicate the individuals by proper collaboration on the diagnostics and clinical care issues within Malaysia and SEA. The HVP SEA node will be established by taking Malaysia as the regional role model in genomic research and diagnostic services especially for the developing countries in SEA. MyHVP is able to assist these countries through human capital development by providing the proper trainings and educations also, increase the public awareness about the importance of genetics and genomics, as main health care contributors.

The Human Variome Project addresses global data sharing on its vision. This vision can be achieved by emphasizing to the four areas of activities for the Human Variome Project, which are set normative functions, behaving ethically, sharing knowledge and building capacity. MyHVP was established on these four foundations, and focused on sharing all information on genetic variations. This will ultimately lead to speedier, better and cheaper diagnosis followed by treatment of genetic disorders as well as better insight into the causes, severity and effect of common disease.

MyHVP Database is not only useful for those who are interested in general/specific mutations or disease specific database such as colorectal cancer, breast cancer or thalassemia; however, it would be useful as the future reference for the other researchers globally.

### Availability

The MyHVPDb is open to access by registering through the website address at http://hvpmalaysia.kk.usm.my/mhgvc/index.php?id=login.

**Project name:** Malaysian Node of the Human Variome Project (MyHVP)

**Project home page:**http://hvpmalaysia.kk.usm.my/

**Operating system:** Windows

**Programming languages:** HTML, CSS, JavaScript, Php, MySQL

**Other requirements:** none

**License:** none required.

## Abbreviations

HVP: Human Variome Project
MyHVP: Malaysian Node of the Human Variome Project database
HTML: Hypertext Markup Language
CSS: Cascading Style Sheet
PHP: Java Script, Hypertext Pre-processor
SQL: Structured Query Language
CGI: Common Gateway Interface
SNP: Single Nucleotide Polymorphism
NCBI: National Center for Biotechnology Information
MAF: Minor Allele Frequency
CNV: Copy Number Variation
SEA: Southeast Asia

## References

[1] Ruangrit U, Srikummool M, Assawamakin A, Ngamphiw C, Chuechote S, Thaiprasarnsup V (2008). Thailand mutation and variation database (ThaiMUT). Hum Mutat.

[2] Tan EC, Loh M, Chuon D, Lim YP (2006). Singapore Human Mutation/Polymorphism Database: a country-specific database for mutations and polymorphisms in inherited disorders and candidate gene association studies. Hum Mutat.

[3] Kleanthous M, Patsalis PC, Drousiotou A, Motazacker M, Christodoulou K, Cariolou M (2006). The Cypriot and Iranian National Mutation Frequency Database. Hum Mutat.

[4] Teebi AS, Teebi SA, Porter CJ, Cuticchia AJ (2010). Arab genetic disease database (AGDDB): a population-specific clinical and mutation database. Hum Mutat.

[5] Meigs JB, Soranzo N (2010). Response to comment on: Soranzo et al. common variants at 10 genomic loci influence hemoglobin A1C levels via glycemic and nonglycemic pathways. Diabetes.

[6] Tabara Y, Kohara K, Kita Y, Hirawa N, Katsuya T, Ohkubo T (2010). Common variants in the ATP2B1 gene are associated with susceptibility to hypertension: the Japanese Millennium Genome Project. Hypertension.

[7] Anney R, Klei L, Pinto D, Regan R, Conroy J, Magalhaes TR (2010). A genome-wide scan for common alleles affecting risk for autism. Hum Mol Genet.

[8] Abdulla MA, Ahmed I, Assawamakin A, Bhak J, Brahmachari SK, Calacal GC (2009). Mapping human genetic diversity in Asia. Science.

[9] Department of Statistics (2007). Malaysia Year Book of Statistics.

[10] Ministry of Health, Malaysia. Health Facts 2009. The most common causes of death in Malaysia. Available at http://www.moh.gov.my/images/gallery/stats/heal_fact/healthfact_L_2009.pdf.

[11] Ainoon O, Cheong SK (1994). Thalassaemia in Malaysia: a strategy for prevention. Malays J Pathol.

[12] Thong MK, Bazlin RI, Wong KT (2005). Diagnosis and management of Duchenne muscular dystrophy in a developing country over a 10-year period. Dev Med Child Neurol.

[13] Goh KJ, Tian S, Shahrizaila N, Ng CW, Tan CT (2011). Survival and prognostic factors of motor neuron disease in a multi-ethnic Asian population. Amyotroph Lateral Scler.

[14] Sinniah D, Narasimha G, Prathap K (1980). Advanced retinoblastoma in Malaysian children. Acta Ophthalmol.

[15] Amini F, Ismail E, Zilfalil BA (2011). Prevalence and molecular study of G6PD deficiency in Malaysian Orang Asli. Intern Med J.

[16] Boo NY, Arshad AR (1990). A study of cleft lip and palate in neonates born in a large Malaysian maternity hospital over a 2-year period. Singapore Med J.

[17] AlAama J, Smith TD, Lo A, Howard H, Kline AA, Lange M (2011). Initiating a human variome project country node. Hum Mutat.

